# Primary posterior optic buttonholing as a new standard-of-care: rationale, technique, advantages, and special indications

**DOI:** 10.3389/fopht.2026.1757735

**Published:** 2026-06-12

**Authors:** Rupert Menapace

**Affiliations:** Department of Ophthalmology and Optometry, Medical University of Vienna, Vienna, Austria

**Keywords:** Anterior capsule polishing, Berger space, capsule tension rings, posterior capsule opacification and laser capsulotomy, posterior optic buttonholing, primary posterior capsulorhexis, refractive stability, rotational stability

## Abstract

**Introduction:**

Posterior capsule opacification (PCO) is still frequent in spite of sharp-edged optic IOLs. With primary posterior capsulorhexis (PPCCC), lens epithelial cells (LECs) may still access the central posterior optic surface. Additional posterior optic buttonholing (POBH) deviates migrating LECs towards the anterior optic surface, thus excluding retro-optical opacification.

**Methods:**

In 2004 the author started investigating POBH as a standard procedure for adult cataracts. 1000 consecutive POBH cases were evaluated and compared to standard in-the-bag IOL placement and sole PPCCC. Additional anterior capsule polishing (ACP) and capsule tension ring (CTR) implantation were also performed in sub-series.

**Results:**

In all eyes, retro-optical opacification was eradicated, and capsular fibrosis largely reduced. CTR implantation facilitated PPCCC with loose zonules or flaccid capsule bags. Postoperative refractive and rotational stability were immediate. No pressure rises or inflammatory response was observed. Retinal detachment and macular edema were not increased.

**Discussion:**

With POBH the IOL optic is placed in Berger´s space outside of instead of in the capsular bag. POBH is controlled, safe, and effective including costs. POBH is compatible with many open-loop IOL models independent of material properties. It avoids dysphotopsia as round-edged optic IOL may also be used. Planned POBH may be converted into sulcus or anterior capsule IOL fixation in case of posterior capsular complications. It provides immediate refractive and perfect rotational stability. POBH improves the performance of trifocal IOLs and facilitates delayed exchange if needed. It combines with Add-On IOLs and translimbal floaterectomy. POBH could therefore replace in-the-bag IOL implantation as standard of care.

## Introduction

Cataract surgery involves two main steps: cataract removal and intraocular lens (IOL) implantation. Coaxial phacoemulsification and foldable IOL injection into an evacuated bag have become standard-of-care procedures. With sharp posterior IOL optic edges, after-cataract and posterior capsule opacification (PCO) rates, as well as YAG laser capsulotomy (YAG-LCT) rates, have been significantly reduced. By adjusting IOL design elements, axial and rotational IOL movement has been minimized, and refractive outcomes have been improved.

## Shortcomings and disadvantages of in-the-bag IOL placement

After-cataract formation with bag-placed, sharp-edged IOLs remains a clinically significant problem: 1. Overlap of the IOL optic by the anterior capsule is a prerequisite for inducing a capsular bend that prevents LEC migration. The overlap should be circumferential and symmetrical. Appropriate centration and sizing of anterior capsulorhexis (ACCC) are not achieved in all cases when performed manually. A scarce or overly large rhexis–optic overlap may result in rhexis retraction and posterior capsule fibrosis, or rhexis phimosis, respectively. An asymmetric overlap may cause IOL decentration and tilt. 2. Posterior optic edges are often not sharp enough to induce effective capsular bending, particularly with hydrophilic IOLs, where hydration blunts the once sharp edge (1–4). 3. With modern one-piece IOLs, sharp edges are inherently not circumferentially continuous. Apart from plate-haptic IOLs, this is particularly true for softer hydrophilic IOLs that require broader haptic-optic junctions for mechanical stability. The so-called enhanced edge beneath such broad junctions is actually a low-level step that does not induce a permanent capsular bend. Full 360°circumferential capsular bending is possible only with three-piece IOLs. Only thread loop haptics feature an optic junction slim enough to be fully bridged by the posterior capsule running beneath it, thus ensuring a continuous sharp bend. 4. The barrier effect of a posterior optic edge, even if exquisitely sharp, wears off over time not only with hydrophilic but also with hydrophobic acrylic IOLs. This occurs because the pressure of a growing Soemmering´s ring may mechanically re-separate the once-fused capsules over time, eventually breaking the collagenous sealing line and reversing the posterior capsular bend along the optic rim. This collagenous sealing is catalyzed by the IOL material and is strongest with silicone and weakest with hydrophilic acrylics. Although low during years 1–3, the PCO rate of a sharp-edged hydrophobic acrylic IOL approached that of the same-design round-edged counterpart after 7–8 years (5, 6). Even with the Acrysof 3-piece hydrophobic IOL featuring an exquisitely sharp posterior optic edge and thread haptics, the barrier effect decayed over 10 years (7). Longer-term YAG-LCT rates vary greatly, with an estimated mean of 20%–30%, or more. 5. Sharp optic edges may induce positive and negative dysphotopsia, which is considered one of the major problems of modern cataract surgery (8). 6. PCO requiring YAG-LCT is often either missed by the patient or underestimated by the ophthalmologist. Patients often fail to consult or have no access to ophthalmologists. PCO develops slowly and is not readily perceived by the patient until the second eye is affected. In developing countries, access to lasers may be difficult or impossible. Therefore, the problem is not only the cost or availability of YAG-LCT, but even more so, the timely awareness and diagnosis of the PCO itself. 7. YAG laser treatment may cause floaters, uveitis, cystoid macular edema, and retinal tears. 8. The cost per eye in the US is approximately 1,000 USD (Care Calculator, Sidecar Health), and the estimated number of YAG-LCT procedures performed per year is 250,000–500,000, summing up to a quarter to half a billion dollars.

## Efficacy of capsular polishing or bending ring implantation as PCO-inhibiting alternatives

Attempts have been made to reduce PCO formation with sharp-edged IOLs. Anterior capsule polishing (ACP) (9) is effective in avoiding fibrosis and thus whitening and shrinkage of the rhexis leaf but increases regeneratory PCO- and YAG-LCT rates due to edge barrier failure. Anterior lens epithelial cells (LECs) transdifferentiate upon contact with the IOL material and start depositing collagen, gluing both capsules together and thus forming a sealing line along the optic rim. ACP removes these anterior LECs and thus the substrate for collagenous capsular sealing, favoring optic edge barrier failure.

The implantation of a sharp-edged capsular bending or distance ring (10) significantly reduced both rhexis leaf fibrosis and regeneratory PCO; however, design improvements were required for full efficiency, which were not implemented.

## Primary posterior capsulorhexis and posterior optic buttonholing

**Figure 1:**
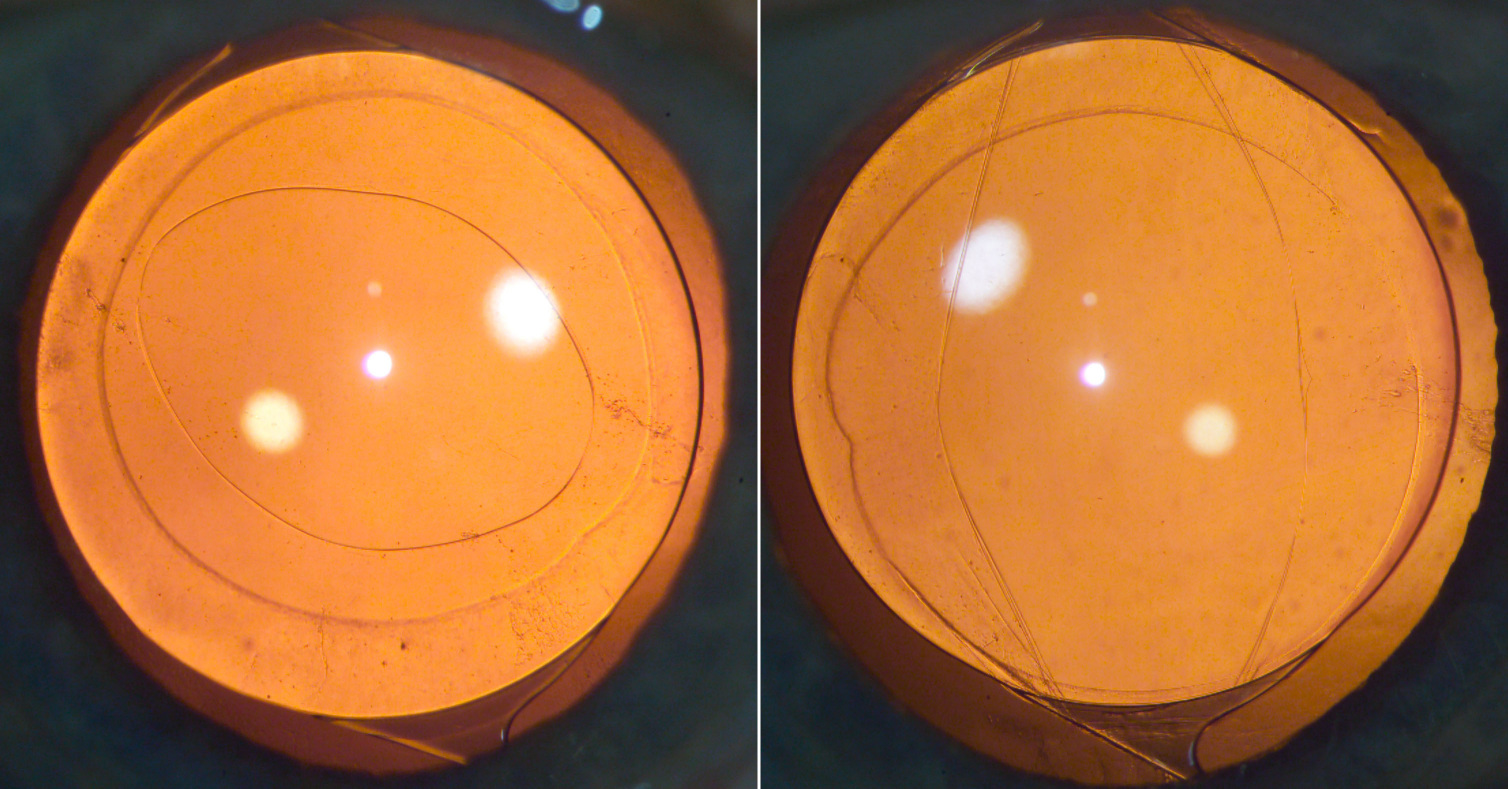
Sole PPCCC on left, and combined POBH on right. Left: PPCCC with bag-placed optic; Right: POBH with optic placed in Berger´s space.

Gimbel and DeBroff (11) first described posterior optic buttonholing (POBH) (**Figure 1**) in a primary posterior capsulorhexis (PPCCC) in pediatric cataract eyes as an alternative to anterior vitrectomy to prevent secondary after-cataract formation. In 2004, the author began to systematically evaluate the efficacy and safety of posterior optic buttonholing (POBH) in adults when used as a standard procedure. The surgical technique and results of the first 500 consecutive cases were published in the Journal of Cataract and Refractive Surgery in 2006 (12), followed by a review of the first 1,000 consecutive cases in Graefes Archives of Clinical and Experimental Ophthalmology in 2008 (13).

## Efficacy and safety of POBH in adults

**Figure 2:**
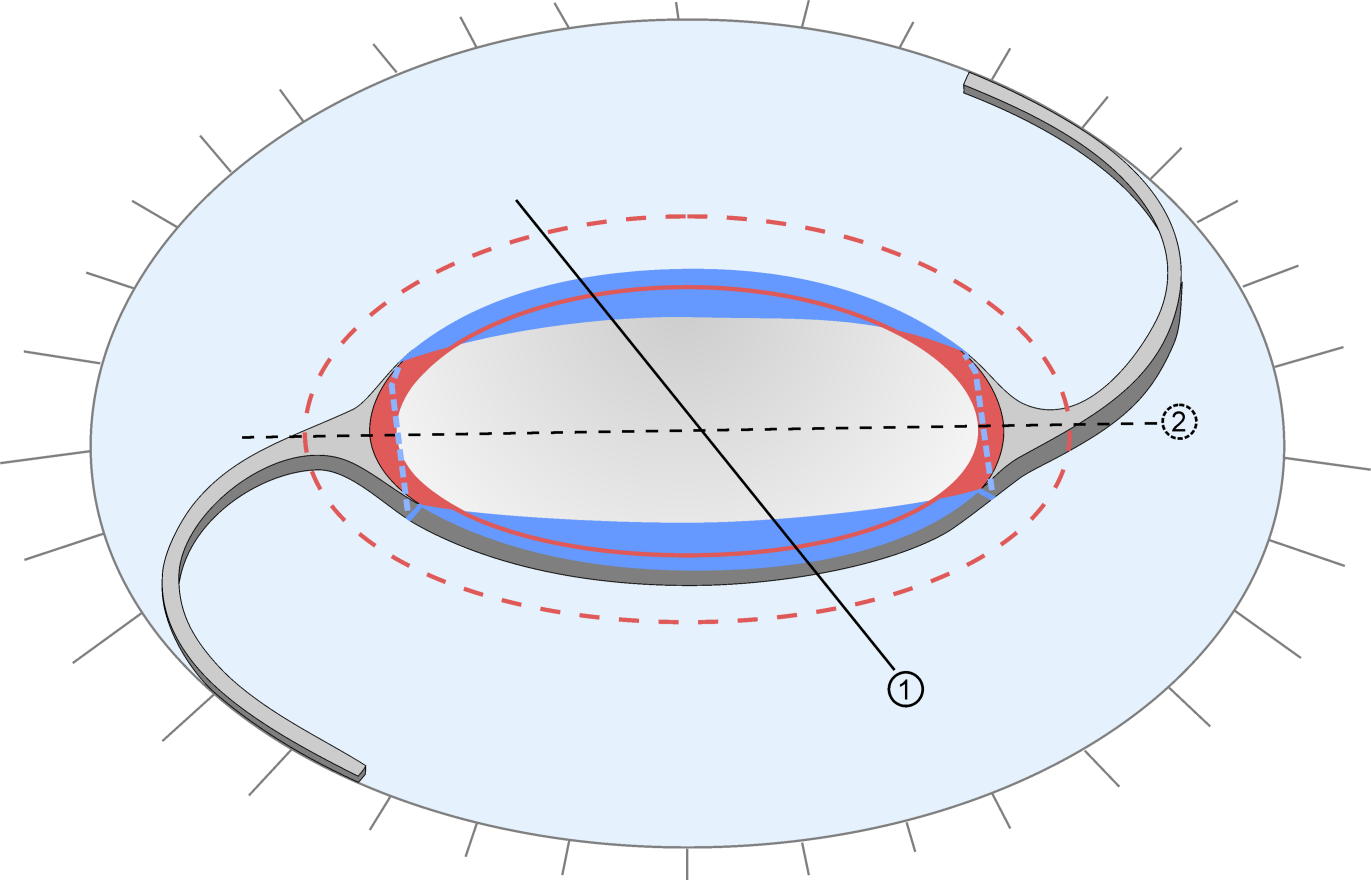
Sandwitched posterior capsule prevents anterior capsule from contacting IOL optic.

**Figure 3:**
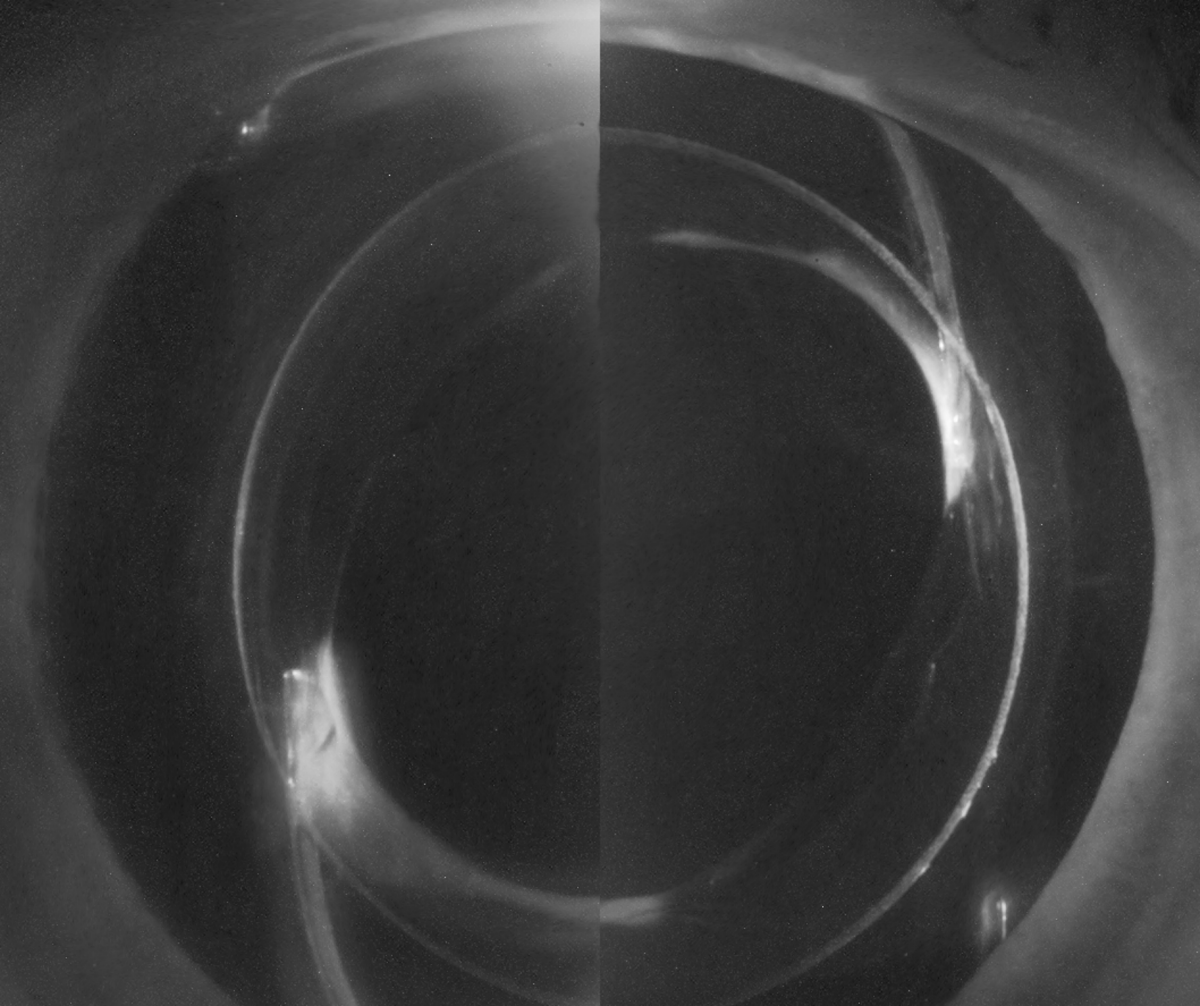
Anterior capsule-IOL contact with consecutive fibrosis confined to area adjacent to haptic-optic junction.

POBH inherently results in the full eradication of PCO by removing the capsular scaffold behind the IOL optic and by redirecting migrating equatorial LECs anteriorly onto the optic surface and towards the anterior chamber. Equatorial LECs are trapped in the sealed capsular fornix or, if not, are exposed to growth-inhibiting agents in the aqueous. The posterior capsule leaf is sandwiched between the anterior rhexis leaf and optic periphery, which prevents the anterior LECs residing on the central areas of the anterior rhexis leaf from establishing contact with the IOL and thus from undergoing myofibroblastic transdifferentiation (**Figure 2**). Except for the area adjacent to the haptic junctions, where the posterior capsule rim undercrosses the haptic and thus allows the anterior capsule to be in localized contact with the optic, fibrosis, and the resulting whitening and shrinkage do not develop, because such contact is a prerequisite for myofibroblastic transdifferentiation to be catalyzed (**Figure 3**).

Safety issues were addressed in prospective randomized intra-individual comparison studies: intraocular inflammation as measured by laser-flare-cell-photometry (14) showed no difference to the standard in-the-bag implantation, as was true for the early postoperative pressure course with and without pressure-lowering drops (15). With regard to postoperative axial movement, the buttonholed optic immediately took its final position, whereas the bag-placed IOL generally exhibited a delayed anterior shift during the first month before finally settling more anteriorly. Consequently, refraction with POBH was stable from the first day onward, while refraction after in-the-bag implantation took a month to stabilize with a tendency towards a myopic shift (16). Macular and retinal complication profiles of PPCCC and POBH were also investigated: OCT-based macular thickness and morphology studies again did not show any differences (17). No cases of clinically significant pseudophakic macular edema (CSPME) were found in the author´s 1,000-case series when excluding eyes and patients with risk factors. In a retrospective study by Scheers et al. (18), there was no case of CSPME in 2,400 eyes during the first 3 months in non-risk cases. The CSPME rate in the subgroup with risk factors was 0.57%, with renal insufficiency, exudative age-related macular degeneration, and retinal vein occlusion as the main risk factors. The 3-month incidence in the total population was 0.29%. In the author´s 1000-eye series with POBH, only two cases of retinal detachment were encountered, one in a young male with high axial myopia 4 months after surgery. A study published by Van den Heurck et al. (19) on long-term retinal complications with the bag-in-the lens (BIL) technique found retinal detachment (RD) rates comparable to or even lower than those reported for standard in-the-bag implantation: In 3,385 BIL cases, the cumulative RD incidence rate was 0.66% after 2 years and 1.17% after 5 years. When excluding eyes with risk factors, the rates dropped to 0.00% and 0.15%, respectively. As with standard cataract surgery, five risk factors were confirmed: male sex, age <60 years at the time of surgery, axial length ≥25.0 mm, a history of contralateral RD, and intraoperative surgical complications. This supports that the controlled removal of the posterior capsule while preserving the anterior hyaloid membrane (AHM) does not increase the incidence of retinal detachment.

## Technique of PPCCC and POBH: pearls and pitfalls

The technique of PPCCC and POBH have been described in detail in previous publications (AAO EyeWiki: Mastering the Posterior Capsule and Optic Capture).

There are a few technical details that deserve attention.

1. With all lens material removed and the posterior capsule cleaned, a cohesive ophthalmic viscoelastic device (OVD) is instilled to tamponade the anterior chamber. When preparing for PPCCC, the capsular bag must not be inflated but instead the posterior capsule flattened out, and the anterior capsule must be gently pushed down to collapse the capsular fornix and thus obtain a common flat “bi-capsular diaphragm.” 2. When initiating the PPCCC, a 30-gauge needle is used to create and elevate a central capsule fold before horizontally perforating it. This is to avoid inadvertent injury to the often closely adjacent AHM. The slit created should be aligned with the cataract incision to allow easy grasping of the edge by the capsular forceps. 3. One edge of the slit is grasped with forceps, and the first quadrant of the PPCCC is performed before starting central viscoseparation of the AHM and posterior capsule. This allows checking the integrity of the central AHM, which may be in close contact with the posterior capsule before starting viscodissection. While gentle OVD injection proceeds, the orifice of the cannula is moved back and forth to avoid backward or forward bulging of the central posterior capsule, which may otherwise cause uncontrolled peripheral extension of the PPCCC. 4. The PPCCC is then continued and completed. The size of the PPCCC should be 4 mm–5 mm. 5. With the PPCCC completed, OVD is injected between its edge and the AHM to circularly expand Berger´s space to approximately *8* mm or even more peripherally, should Wieger´s ligament readily detach or be already partly or fully detached. This creates ample space for safe injection and buttonholing of the IOL. 6. As an IOL implant, a 6 mm optic open-loop acrylic IOL with a continuous-transition haptic-optic junction and stepped-vaulted haptics (HOYA AF-1) was used as the preferred implant for the study. However, today´s preferred single-piece acrylic IOLs can also be easily and safely used, preferably those with a slim or notched haptic-optic junction. 7. For OVD removal from the anterior chamber at the end of surgery, the incisions—particularly the paracentesis openings—must be carefully hydrated, and a *sleeved coaxial* I&A tip is used and quickly retracted when OVD aspiration is completed. Otherwise, the chamber may flatten due to leaking aqueous and the bulging IOL unbutton. Needless to say, no effort is made to aspirate the OVD from behind the IOL since it is trapped by the hermetical IOL-capsule-diaphragm and thus will never access the anterior chamber to cause an IOP rise. Any such unnecessary effort is strictly forbidden because it would jeopardize the integrity of the AHM and risk AHM damage with vitreous prolapse (https://youtu.be/U8DZ4L1KQhQ) (**Figure 1**).

The question of whether PPCCC can be performed with an IOL already implanted arises. To allow for forceps access to the capsule, the edge of an already implanted IOL optic must be lifted. The haptics of the tilted and decentered IOL exert asymmetric traction forces on the posterior capsule. In addition, it is difficult to flatten the posterior capsule. Consequently, PPCCC is less controlled with the IOL in place. Under these circumstances, if the PPCCC runs out towards the periphery or if the AHM is damaged, the presence of the IOL would make remedy more difficult and riskier. Therefore, performing PPCCC first and IOL implantation second is definitely preferable.

## Combining POBH and capsule tension ring implantation with an oversized capsular bag and/or defective zonular apparatus

Both conditions result in a lax capsule, which makes designing an appropriate PPCCC difficult. This may become apparent when performing the ACCC or during lens workup and cortex aspiration. If so, a capsule tension ring (CTR) is inserted just before initiating the PPCCC. With the flat bi-capsular diaphragm already created, the leading eyelet is threaded into the collapsed fornix. The CTR radially expands the capsular bag and equally redistributes the traction forces exerted during the PPCCC procedure as well as by oversized or overly rigid IOL haptics. Bimanual insertion is recommended to minimize zonular stress and the risk of fornix perforation, as it enables the leading eyelet of the CTR to engage and then glide along the equator at an acute angle. Any capsular entanglement is immediately signaled by radiating stress folds and the whipping back of the CTR while gently advanced by repeatedly re-grasping the trailing CTR section with capsule forceps. To avoid friction and dragging of the rhexis rim, the CTR glides through a Y-spatula inserted through a side-port and positioned proximally to the rhexis rim crossover. Finally, the trailing end and eyelet are leveraged above the rhexis rim into the capsular fornix (https://www.youtube.com/watch?v=b7ZVHPrsSQU, https://youtu.be/3Au1WsJgL8Y).

If laxity of the capsular bag is perceived only when the PPCCC has already been initiated, a CTR can still be inserted at any time during or after the PPCCC procedure. The atraumatic bimanual insertion technique described above is particularly helpful and recommended in these circumstances. The CTR can even be inserted with both IOL haptics already residing in the bag to transform an ovalized into a round anterior and posterior capsulorhexis before buttonholing the optic.

## Combining POBH and anterior capsule polishing for possible delayed IOL exchange

Secondary exchange may become necessary in pediatric eyes or in eyes with presbyopia-correcting IOLs. With in-the-bag IOLs, delayed surgical re-division of fused and shrunken capsules may be difficult and risky. POBH per se inherently reduces fibrosis because of the above-described sandwich effect of the posterior capsule. However, local fibrosis may develop near the haptic–optic junctions. Additional anterior capsule polishing (ACP), with specific consideration of these capsule areas completely prevents fibrosis, rendering delayed reopening of the capsular bag fornix, as required for IOL exchange, even easier and atraumatic (https://www.youtube.com/watch?v=bX2WkVCnDew). Unlike after in-the-bag placement of IOLs, ACP with POBH does not negatively impact after cataract formation.

## Role of the anterior capsule leaf and impact of anterior capsulorhexis sizing and optic overlap

With POBH circumferential overlap of the IOL optic by the anterior capsule leaf is not required, as the mechanism of capsular fusion, bending, and sealing along the optic rim to inhibit LEC migration is replaced by redirecting the LECs to the anterior surface of the buttoned-in optic. An undersized or decentered PPCCC is much less critical than an undersized or decentered ACCC with standard in-the-bag implantation. However, a well-sized and centered ACCC can be used as a ruler while performing the PPCCC. With adequate experience and aids for adequate PPCCC sizing, a very large ACCC diameter may be chosen to decrease the anterior LEC population and avoid direct optic contact where the PPCCC undercrosses the haptic junction following POBH. Thus, fibrosis is excluded even without additional ACP. If a minimum diameter of the PPCCC cannot be guaranteed, a no larger than 5 mm ACCC allows alternative anterior rhexis fixation of the IOL should the PPCCC run too large. Secondary enlargement of a smaller ACCC by forceps after successful POBH is still an option.

## Escape techniques with oversized PPCCC or anterior hyaloid membrane

A frequently asked question relates to common complications and their management strategies. When an unplanned posterior capsule injury occurs during phaco or I&A, the tear may usually not be readily converted into an adequately sized and centered PPCCC, and the anterior hyaloid membrane (AHM) is often damaged, with vitreous protruding. When performed as a planned PPCCC procedure after adequate flattening of the posterior capsule and viscoseparation of the AHM, and following the surgical regimen step by step as described, sizing of the PPCCC with a flattened posterior capsule is easy to control, and injury to the AHM is unlikely. Complications are very rare and are easy to manage. For a skilled and dedicated surgeon, a learning curved of 50–100 cases should be sufficient.

Management of PPCCC and AHM complications, if they occur, is as follows:

1. Oversized PPCCC: As mentioned, appropriate sizing of the PPCCC (4 mm–5 mm) is crucial. The ACCC can be used as a ruler to help guide the PPCCC along and preferably slightly inside the anterior rhexis rim to ease IOL haptic insertion. Assessing the actual diameter of a manual anterior rhexis by a measuring spatula, digital projection, or creating a perfectly sized capsulotomy with a femtosecond laser facilitates appropriate sizing. In case of a PPCCC that is still too large or evasive, a 6.5 mm optic IOL (HOYA AF-1VA65BB 3-piece, or Ophthalmo Pro Proming Big Optic ALD single-piece IOL) can be used. Alternatively, a 3-piece IOL with thread loops can be positioned in the sulcus and buttoned into the anterior rhexis (anterior optic buttonholing). If not available, the optic of a bag-fixated open-loop IOL may also be buttonholed through an intact ACCC (reverse optic buttonholing). Both options are applicable with a larger posterior capsule defect that cannot be converted into an appropriate PPCCC.
2. Anterior hyaloid membrane injury: Even with an appropriate PPCCC technique, AHM injury may occur in individual cases. Berger’s space may be very shallow, if not absent. Even after transzonular hydroseparation (20), the AHM may remain attached to the central posterior capsule and thus be inadvertently punctured when initiating the PPCCC. Delaying viscodissection of the AHM before a quadrat flap has been created as described above is helpful for assessing and preserving AHM integrity. With unnoticed AHM puncturing, the OVD may be instilled into the vitreous body or a “hyalo-capsulorhexis” may be created. If so, the vitreous can either be pushed back by OVD or removed by limited vitrectomy when prolapsing and the IOL safely implanted and buttonholed. No cases of vitreous entrapment or retinal complications have been reported to date.

## Suitable and unsuitable eyes

POBH can be used with all eyes featuring adequate mydriasis and zonular stability. Eyes with a normal-sized anterior segment and well-dilatable pupils are best suited for beginners. Although accessible for POBH after insertion of pupil-dilating rings, eyes with small pupils may be better candidates for standard in-the-bag IOL implantation, particularly for less experienced surgeons. Eyes with pseudoexfoliation and generalized progressive zonular weakness are best served with combined anterior and posterior optic buttonholing (“Hyaloid-sparing double capture or HSDC introduced by Lisa Arbisser) of a 3-piece IOL with thread loops positioned in the sulcus. Marfan eyes or those with congenital zonular or lens colobomas may be considered after capsular tension ring or ring segment insertion but may be better served by standard surgery.

Although eyes with normal axial length are preferable, POBH is also suitable for long and short eyes. In eyes with an abnormally large anterior segment (anterior makrophthalmus), adequate estimation and sizing of the PPCCC may be more difficult. Calipers or projected rings are helpful for avoiding oversizing.

Vitreous liquefaction or posterior vitreous detachment have no relevant impact. The proximity of the AHM to the posterior capsule is a key parameter. OCT scanning is currently unable to discern the AHM and Berger’s space preoperatively, especially through denser cataracts. Intraoperative OCT imaging after cataract removal is feasible (20), but not helpful as a routine. Visual indicators for a pre-existing AHM detachment are a flaccid posterior capsule and emulsified lens particles circulating behind it when the nucleus and central cortex have been removed. If no visual hints as to the interspace are present, utmost care is necessary while creating and perforating the central posterior capsule fold to initiate the PPCCC, because the AHM may be closely attached to the central posterior capsule.

Ocular comorbidities, such as glaucoma or diabetes, are not considered contraindications.

## Lens designs compatible with POBH and biometric adjustment

POBH is compatible with any IOL design that features an open loop with a not-too-broad optic junction. Thus, most of the currently used hydrophobic single-piece IOLs can be used. The softer hydrophilic IOLs require thicker or fenestrated loops with broader haptic–optic junctions for sufficient mechanical stability, which may make some models less suitable or even unsuitable. There is no question that plate-haptic designs cannot be buttoned in.

With POBH, the IOL optic settles slightly more posterior than after standard in-the-bag placement. For modern single-piece IOLs, pertinent data have not yet been collected, but the refractive impact is considered to be small. Adjusting the target refraction by a quarter of a diopter with an IOL-power >22.0 diopters, and half a diopter with a power >26.0 diopters, compensates for the slight loss of refractive IOL power.

## Advantages of POBH over sole PPCCC

PPCCC without buttonholing is efficient, as it removes the central posterior capsule, which serves as a scaffold for migrating LECs. However, LECs may alternatively grow on the AHM or on the posterior optic surface and form a contiguous cell layer that may partially or totally occlude the PPCCC opening (21). Due to their biocompatibility, hydrophilic IOLs are particularly prone to re-occlusion. In addition, with sole PPCCC, the OVD left behind the IOL optic may gain access to the anterior chamber and cause pressure elevations or spikes (22). In cases of AHM injury, prolapsing vitreous may be entangled between the optic and the capsule. All this is prevented by buttonholing the optic into the PPCCC opening, thereby ensuring a permanent hermetic seal and separation of the anterior and posterior eye compartments. Sole PPCCC is an option with plate-haptic IOLs, where the jagged posterior capsule rim may extend peripherally and the anterior chamber may deepen following YAG laser capsulotomy, causing a hyperopic shift. Also, firm PPCCC–optic attachment and optic surface hydrophobization help counteract reclosure of the PPCCC opening by LEC outgrowth (23).

## Additional value of POBH

Considering the still high PCO and YAG laser capsulotomy rates with modern sharp-edged acrylic IOLs, PPCCC with POBH should be used as a standard of care in all cataract patients with a sufficiently wide pupil and an intact zonular apparatus. Additional values are: 1. Immediate postoperative refractive stability, which allows prescription of final glasses and IOL power adjustment in sequential cataract surgery of the partner eye within a short time. 2. POBH provides total postoperative rotational stability, independent of the IOL loop design and diameter, making it ideal for toric IOL implantation. 3. POBH is the ideal technique for *presbyopia-correcting IOLs*, particularly trifocals, since it avoids additional straylight by permanently excluding the formation of thin, homogeneous LEC layers, which may be hardly visible at the slit lamp while significantly degrading optical performance. 4. Since the mechanism of action is not based on capsular bending and is thus independent of the optic edge design, round-edged optics with edge profiles designed to minimize disturbing light phenomena can be used with POBH. Therefore, dysphotopsia—rated as the number one problem in modern cataract surgery—is totally avoided. 5. The buttoned-in IOL optic and capsule leaves form a planar common diaphragm, which provides ample distance to the iris up to the very periphery and thus ample space for add-on IOLs with minimal risk of iris chafing.

## Special indications for POBH: presbyopia-correcting IOLs and floaters

There are two important settings in which this technique should be considered:

1. Delayed exchange of presbyopia-correcting IOLs: The main obstacle to the more widespread use of multifocal IOLs (MIOLs) and extended-depth-of-field (EDOF) IOLs is the difficulty of assessing visual performance and tolerance in a given patient. Postoperative photic phenomena often resolve due to neuroadaptation. However, adaptation may take 3 months or even longer. During this time, the process of capsular healing is complete, and collagenous sealing is finalized. This may make reopening of the capsular fornix difficult and risky, if not unfeasible. With POBH, fibrosis is limited to the small areas where the posterior capsule undercrosses the haptic junction. Additional anterior capsule polishing, as described above, fully prevents fibrotic changes and is therefore particularly recommended. With the elasticity of the capsular diaphragm thus preserved, the optic can be buttoned out and the IOL exchanged easily and safely at any time after surgery. The technique is straightforward and well controlled: The anterior chamber is filled with cohesive OVD to stabilize the lens–capsule diaphragm. Then, the tip of the OVD cannula is directed beneath the edge of the IOL optic. The AHM is gently detached, and Berger’s space is reformed. The IOL can now be safely exchanged, either by cutting or refolding, and replaced with the IOL of choice.
2. *Floaterectomy*: Floaters may be so disturbing that removal is indicated. Floaters can be particularly annoying with MIOLs. YAG laser vitreolysis is popular but must be viewed critically, particularly when floaters are large and located close to the retina. Removal by pars plana vitrectomy is the appropriate approach but carries the risk of creating peripheral retinal tears, especially around the sclerotomies. A PPCCC allows transpupillary access to the posterior segment, thereby allowing safe translimbal floaterectomy. The technique is again straightforward and well controlled: With the PPCCC completed and the AHM fully detached by OVD, a 23- or 25-gauge vitrectome is inserted through a paracentesis and advanced through the PPCCC. An I&A infusion handpiece is introduced through a second paracentesis and positioned above the PPCCC. Vitrectomy is performed by first removing the anterior and core vitreous, and then the deeper and peripheral vitreous as required. Retroillumination provides excellent visualization. By rotating the globe and extending the PPCCC with the two instruments, ample access is obtained. With vitrectomy completed, the vitrector is retracted, followed by the infusion handpiece, while the anterior segment is still tamponaded with cohesive OVD. A cannula is advanced through the OVD bolus, and the vitreous cavity is filled with balanced salt solution until the globe is tonicized. The IOL is then injected and buttonholed as usual. A total of 67 eyes have been operated on with this technique over the last 15 years, all with successful removal of the floaters and no retinal complications.

## Alternative techniques to POBH

Double capture (“Hyaloid sparing double capture”, HSDC): With this modification of POBH, first described by Lisa Arbisser in 2014, the IOL haptics are positioned in the sulcus and the optic is subsequently buttoned through both the anterior and posterior capsulorhexis openings. This provides stable IOL fixation and is primarily intended for pseudoexfoliation (PEX) eyes with preexistent or progressive zonular weakness. While with POBH the remaining anterior capsule leaf settles on the peripheral IOL optic and posterior capsule, HSDC firmly attaches both capsules, thus securely sealing the capsular fornix. As a Soemmering ring forms, regenerates are trapped in the capsular equator with HSDC. While they may still access the anterior chamber, peripheral pearl formation after POBH is minimal because of the proliferation-inhibiting effect of the aqueous.

HSDC is a promising concept to ensure long-term stability of IOLs in eyes with, or at risk of, future zonular instability. However, there is one downside compared with POBH: with both POBH and HSDC, the IOL optic is centered by the haptics. With POBH, the haptics reside in the capsular fornix, while with HSDC, the haptics are supported in the sulcus. The haptics must be wide and rigid enough to provide adequate centration. Haptic contact to vascularized uveal tissue may destabilize the blood–aqueous barrier, particularly in prone eyes such as PEX eyes. A modified IOL design has been devised by the author to minimize such contact and irritation, but is not yet available.

**Figure 4:**
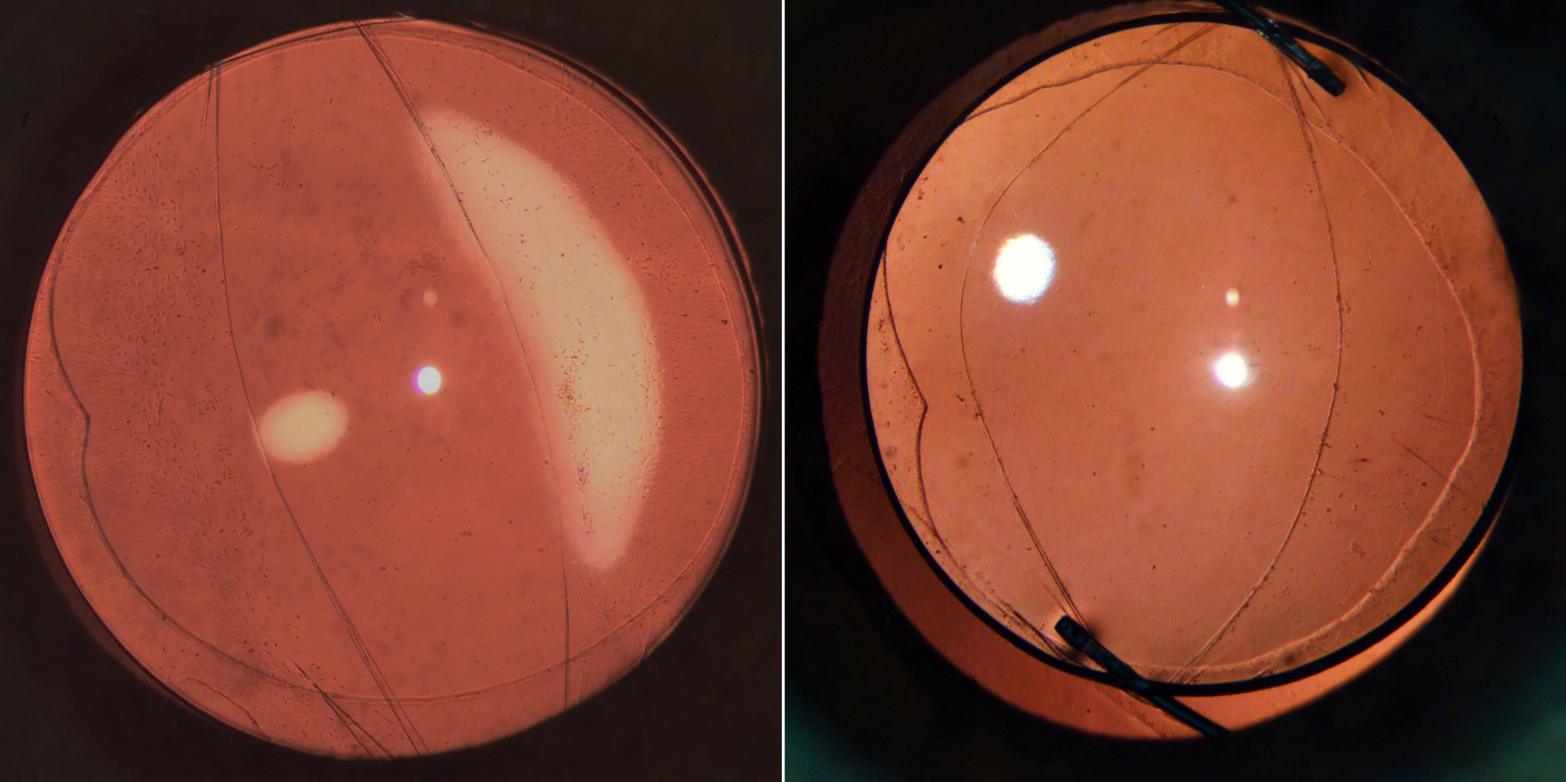
Left: Posterior capsule elasticity still allows optic buttonholing in very small PPCCC: Right: Haptics residing in fornix center optic in capsular bag independent of PPCCC centration.

Bag-in-the-lens (BIL): This concept was introduced by Marie-José Tassignon in the early 2000s (24). A large number of BIL eyes have accumulated over the years. Special CTRs have been designed for eyes with zonular deficiencies. Numerous studies have been conducted and published. Limitations and obstacles impeding the widespread use of this wonderful technique include: 1. the need for a special IOL that cannot be alternatively fixed should capsular problems arise after implantation, and 2. The requirement for perfect sizing and centration of both the anterior and posterior capsulorhexis. Larger incisions are required for controlled implantation, and rulers or lasers are needed to achieve appropriately sized capsulorhexis openings. POBH uses standard open-loop IOLs. These allow: 1. the use of standard devices for perfectly controlled implantation through mini-incisions; 2. Optic buttonholing is still feasible and autocentration guaranteed in both undersized and decentered rhexes openings (**Figure 4**); 3. alternative fixation by anterior or reverse optic buttonholing in case of posterior rhexis or capsule problems.

## Femtosecond laser posterior capsulotomy for PPCCC, POBH, and related techniques

Femtosecond lasers (FSLs) create perfectly sized and centered capsulotomies by arranging micro-explosions side by side in the target plane. As with anterior capsulotomies, posterior capsulotomies of any desired size, shape, and centration can be formed without the use of needles or forceps. This is preferably performed when cataract surgery is completed. The suction cup is redocked to the eye, and the laser is focused on the posterior capsule under OCT guidance.

However, a prerequisite for effective and safe FSL capsulotomy is an adequate distance between the central posterior capsule and both the IOL optic and the anterior hyaloid membrane (AHM). Otherwise, the optic may be pitted, or the AHM and anterior vitreous may be incised. Central adhesions to the AHM may prevent the excised capsular button from moving out of the visual axis.

In a consecutive series of 180 eyes, the author conducted OCT measurements to determine the incidence of preexisting spontaneous AHM detachment at the conclusion of cataract surgery and after forced transzonular hydroseparation (TZHS). With the IOL implanted in the bag and the OVD aspirated from behind the IOL, a first redocking was performed, and the retrooptical space was scanned with OCT. Central, intermediate, and peripheral distances between the posterior capsule and the AHM were documented. If the AHM was not completely detached, TZHS was performed, followed by a second redocking and a second scan. If AHM detachment had increased but was not yet complete, another attempt at TZHS and a scan were made. Finally, diluted triamcinolone was used to visualize the interspaces between the posterior capsule and the AHM. The results were as follows (R. Menapace: Intraoperative OCT imaging of Berger’s space: Findings and clinical significance. Presented at the 36th International Congress of the German Ophthalmic Surgeons, 2024, Nuremberg): At the conclusion of cataract surgery, 18% of eyes showed complete AHM detachment, 27% showed partial detachment, and 55% remained fully attached to the posterior capsule. After the first TZHS effort, the percentages were 35% (+17%), 23% (−4%), and 42% (−13%). After a second TZHS effort with triamcinolone staining, the AHM was fully detached in 43% (+8%), partly detached in 21% (−2%), and still fully attached in 36% (−6%). Thus, the efficacy of TZHS to fully detach a partly or non-detached AHM dropped from an additional 4% to 2% and 13% to 6% after the first and second efforts, respectively. Thus, if full AHM detachment is considered a prerequisite, safe FSL posterior capsulotomy would have been possible in approximately one out of five eyes. By TZHD, which is unlikely to become a routine procedure, this percentage could be doubled to approximately two out of five eyes, or a maximum of half of the cases when performed twice. Our results suggest that FSL posterior capsulotomy is currently not an alternative to the well-controlled manual PPCCC technique, which can be performed in almost any eye. A surgical video showing POBH with anterior capsular polishing and CTR implantation is available at https://youtu.be/UT-8-Ap7tEo.

## Conclusion

Standard in-the-bag surgery stops halfway. Adding PPCCC with POBH fully eradicates PCO, independent of optic edge design, IOL material, or anterior capsule–optic overlap. The complete and permanent eradication of retrooptical opacification reduces healthcare costs by eliminating the need for YAG-CT and avoids its associated complications. Furthermore, capsular fibrosis and its adverse effects are prevented.

The procedure is controlled, safe, and relatively quick to learn for a skilled and dedicated surgeon. Complications are rare and can usually be managed with standard techniques. PPCCC and POBH can be readily converted to sulcus or anterior capsule IOL fixation in cases of posterior capsular complications. POBH uses standard IOL models, avoids dysphotopsia when used with round-edged optic IOLs, and provides immediate refractive and rotational stability. It improves the performance of trifocal and other presbyopia-correcting IOLs and facilitates delayed IOL exchange. It can be combined with anterior capsule polishing or a large ACCC to counteract fibrosis without interfering with posterior capsular opacification prevention. It provides additional space for add-on IOL implantation and facilitates translimbal floaterectomy when combined procedures are required. Therefore, PPCCC with POBH should be considered a standard of care for routine cataract surgery. Instead of the capsular bag, the IOL optic should be positioned in Berger’s space.

## Data Availability

The original contributions presented in the study are included in the article/supplementary material. Further inquiries can be directed to the corresponding author.
